# Detecting peroxiredoxin hyperoxidation by one-dimensional isoelectric focusing

**DOI:** 10.1007/s41048-015-0007-y

**Published:** 2015-08-21

**Authors:** Zhenbo Cao, Neil J. Bulleid

**Affiliations:** Institute of Molecular, Cellular and Systems Biology, College of Medical Veterinary and Life Sciences, Davidson Building, University of Glasgow, Glasgow, G12 8QQ UK

**Keywords:** Hyperoxidation, Peroxiredoxin, Isoelectric focusing

## Abstract

The activity of typical 2-cys peroxiredoxin (Prxs) can be regulated by hyperoxidation with a consequent loss of redox activity. Here we developed a simple assay to monitor the level of hyperoxidation of different typical 2-cys prxs simultaneously. This assay only requires standard equipment and can compare different samples on the same gel. It requires much less time than conventional 2D gels and gives more information than Western blotting with an antibody specific for hyperoxidized peroxiredoxin. This method could also be used to monitor protein modification with a charge difference such as phosphorylation.

## Introduction

Peroxiredoxins (prxs) play major role in removal of H_2_O_2_ in cells (Wood et al. 2003b). In mammalian cells, there are four typical 2-cys prxs (PrxI, II, III and IV). They are located in different subcellular compartments: PrxI and II in the cytosol, PrxIII in the mitochondrial, and PrxIV in the ER. They are characterized by the presence of 2 conserved cysteines that both are important for the reaction cycle. In one reaction cycle, the peroxidatic cysteine reduces H_2_O_2_ and becomes sulfenylated. The cysteine sulfenic acid then forms a disulfide bond with the resolving cysteine from an adjacent protomer. The reaction cycle is completed with the reduction of the disulfide bond by a member of the thioredoxin family of proteins. If the H_2_O_2_ concentration is high enough, however, the cysteine sulfenic acid can be further oxidized to sulfinic acid or even sulfonic acid before it can form a disulfide bond with the resolving cysteine. This is called peroxiredoxin hyperoxidation and leads to enzyme inactivation. In the presence of a rapid recycling system and sufficient H_2_O_2_, the peroxidatic cysteine can be fully hyperoxidized very quickly (Yang et al. 2002). Prxs hyperoxidation has been suggested to play crucial regulatory roles in different pathways (Day et al. 2012; Edgar et al. 2012; Turner-Ivey et al. 2013; Wood et al. 2003a). Therefore, a simple assay to monitor Prxs hyperoxidation is much needed.

Currently hyperoxidation of prxs is either detected by the use of an antibody that specifically binds to the hyperoxidized form, or by two-dimensional electrophoresis. The antibody was raised to a peptide that spans a conserved region around the peroxidatic cysteine of PrxI to PrxIV. As a consequence, it recognizes all forms of hyperoxidized 2-cys prxs (Woo et al. 2003). This method is quick and sensitive but may not be able to distinguish different prxs and is non-quantitative in which it cannot determine the fraction of the total prx that has become hyperoxidized. Two-dimensional electrophoresis can overcome these obstacles. The first dimension, isoelectronic focusing (IEF), separates proteins based on their isoelectronic point. Because hyperoxidized prx has a more negative charge, it will focus at a more acidic point, separates from the non-hyperoxidized form. Individual prx hyperoxidation states can be monitored by Western blotting with protein specific antibodies. The level of hyperoxidation can also be determined this way by quantifying the intensity of different spots. However, this method is labor and time consuming, and different samples have to be compared between different gels which potentially introduce experimental variation. A non-reducing SDS gel system has also been used to monitor the redox state of 2-cys prxs based on the monomer dimer interconversion (Cox et al. 2010). The authors also used this method to measure the extent of hyperoxidation as the hyperoxidized cysteines are not able to form disulfide bonds and therefore become a monomer. This assay has the advantage of monitoring individual 2-cys prxs, comparing different samples on the same gel and easy to setup. Nevertheless, it is difficult to distinguish fully reduced and fully hyperoxidized prxs as they are both monomers. The same problem applies to the dimers because they can be oxidized or partially hyperoxidized prxs. Mass spectrometry has been used to directly measure mass increase caused by hyperoxidation. However, quantification of the level of the hyperoxidation is absent.

## Overall description of the method

Here we report a simple assay to monitor Prxs hyperoxidation by one-dimensional IEF gels. This assay is easy to setup and only uses standard lab equipment. It includes the following steps: Sample treatment → Acetone precipitation → Resuspend sample → Isoelectric focusing → Soak gel with SDS → Transfer and blot.

## Result

**Fig. 1:**
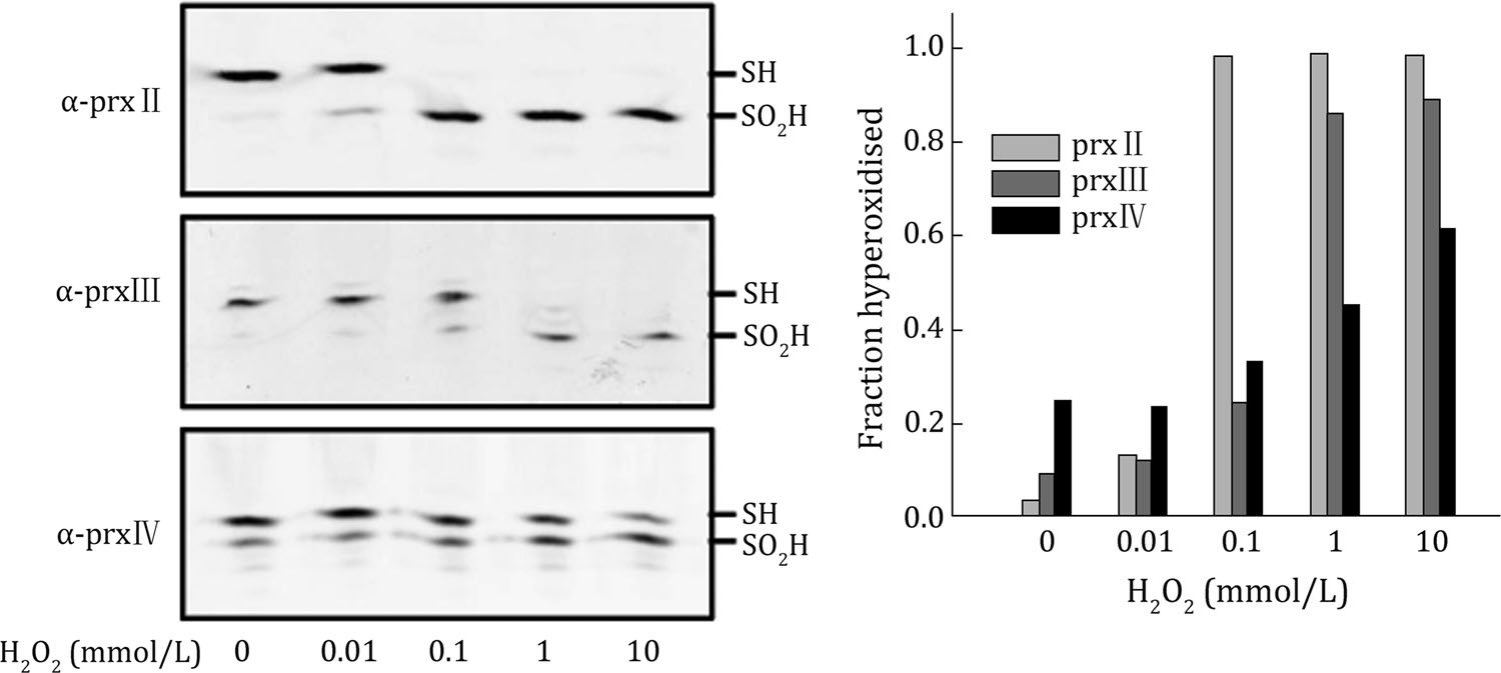
1D IEF assay for Prxs hyperoxidation in Hela cells. Hela cells were treated with different concentrations of H_2_O_2_ for 10 min. Cell lysates were prepared as described in method and resolved on a one-dimensional IEF gel with pH 4–6 ampholytes. Samples were then transferred to nitrocellulose and immunoblotted with PrxII(*α*-prxII), PrxIII (*α*-prxIII), and PrxIV (*α*-prxIV) antibodies. The acidic, hyperoxidized form of prxs (SO_2_H) is separated from the unmodified form (SH). The fraction of hyperoxidized prxs was calculated and plotted

Hela cells grown in 6-cm dishes were treated with different concentrations of H_2_O_2_ for 10 min at 37 °C. To prevent further oxidation, *N*-ethylmaleimide (NEM) was added before cell lysis. Because the isoelectric point of PrxII, PrxIII, and PrxIV are all around 5.5, gels were prepared with ampholytes in the pH range of 4–6. After sample preparation and 1-D isoelectric focusing, proteins were transferred onto a nitrocellulose membrane and blotted with different prx antibodies (Fig. 1). Clear differences can be seen when cells were treated with 0.1 mmol/L H_2_O_2_. PrxII became fully hyperoxidized while the other two prxs were slightly modified. When H_2_O_2_ increased to 1 mmol/L, PrxIII also became fully hyperoxidized. PrxIV was only moderately hyperoxidized even with 10 mmol/L H_2_O_2_. The quantification clearly demonstrated the different sensitivity to H_2_O_2_ treatment of Prxs located in different intracellular compartments. This assay was completed in 2 days.

## Discussion

The assay we reported here took the advantage of isoelectric focusing from conventional 2-D gels but using a 1-D SDS-PAGE setup. Under this setup, multiple samples can be separated on the same gel by their charge differences. We used this assay to study sensitivity difference towards H_2_O_2_ between different prxs in different subcellular compartments simultaneously (Cao et al. 2014). We demonstrated this assay can easily compare multiple samples directly on the same gel, thereby reducing variability between different gels. The extent of hyperoxidation of individual Prxs was also determined by quantifying the hyperoxidized or unmodified forms. The differences between different Prxs suggested that the recycling system for PrxIV in the endoplasmic reticulum is less efficient than for other 2-cys prxs in other subcellular compartments.

This method can easily apply to other approaches looking for protein modification with a charge difference, such as phosphorylation. Moreover, whether the modification occurred can be determined as well as the extent of the modification. The flexibility of different ampholyte pH ranges makes it adaptable to any specific protein of interest. The isoelectric point of the protein of interest, the charge of the modification, and the extent of the band shift of the modification need to be considered in the choice of ampholytes.

## Methods

### Reagents

*IEF sample buffer* (7 mol/L urea, 2 mol/L thiourea, 2% CHAPS, 0.8% ampholytes pH 4–6, 50 mmol/L DTT, 4% glycerol, 0.02% bromophenol blue): The buffer can be stored in aliquots at −20 °C.

*Gel solution* (9 mol/L Urea, 1% CHAPS, 8% acrylamide/bis-acrylamide, 0.4% ampholyte pH 4–6).

*Upper chamber buffer* (25 mmol/L Tris base): The pH of these buffers is not adjusted after dissolving into water. The pH of Tris solutions should be around 10.5.

*Lower chamber buffer* (10 mmol/L phosphoric acid).

### Equipments

PowerPac high voltage power supply (Bio-Rad), Mini Vertical Unit electrophoresis apparatus (Hoefer), and Roller mixer (Stuart) were used.

### Procedures

#### Sample preparation

Protein samples must be free of other charged molecules before loading onto the gels. Use the following procedure for protein isolation:

Five volumes of ice-cold acetone were added to the cell lysate and incubated for at least 1 h at −20 °C. The protein precipitated is then isolated by centrifugation (10,000 *g* for 10 min at 4 °C) and washed with 80% ice-cold acetone. The precipitate is isolated by centrifugation again and the pellet is air-dried. The pellet is resuspended in 50 μL IEF sample buffer. The protein is allowed to dissolve in the IEF sample buffer for at least 1 h at room temperature on a roller mixer. Samples are incubated at 30 °C for 10 min before load onto the gel.

#### 1-D isoelectric focusing

1. Freshly prepare 10 mL gel solution per gel and mix on the roller mixer for 2 h at room temperature. Once dissolved, add APS to 0.1% *w*/*v* and TEMED to 0.1% *v*/*v*, and cast gels with combs on top.
2. After the gel polymerizes, clean away all excess urea from the outside of the gel. If gels are not used immediately, wrap them tightly in plastic wrap and store at room temperature. When ready to use, place gels in running tank. We use the Mini Vertical Unit electrophoresis apparatus from Hoefer. Use Tris base in the upper chamber and phosphoric acid in the lower chamber. Fill the upper chamber to above sample wells and check for leaks. Then fill the lower chamber just to cover the bottom of the gel. Load samples (normally 10 µL) directly into the wells. Leave a couple of wells on either sides of the gel empty.
3. Run the gel at 1000 V for 5 h. Set the current limit to 2 mA per gel using the Bio-Rad high voltage power supply. At the end of the run, the voltage should be 1000 V and current should be less than 1 mA per gel. Top up the upper chamber during the run if needed. Try not to run overnight so as to monitor any leaking that may occur.
4. Remove the gel from the glass plates and wash for 5 min in deionized H_2_O (this step and all subsequent steps should be performed with gentle shaking as for a Western blot). The gel is ready for the Coomassie staining. For Western blotting, wash the gel in transfer buffer for 20 min. The gel is now ready for transferring and blotting with specific peroxiredoxin antibodies.

### Troubleshooting

Sample bands may not be straight if current is too high. Set current as the limiting factor during focusing and also avoid loading samples on the outside wells of the gel. If separation is not ideal, change ampholyte pH range based on isoelectric point of protein of interest. If sample has not transferred to nitrocellulose properly, make sure there is a good contact between gel and membrane in the transfer cassette.
